# Hepatitis E Virus Genotype 3 in Humans and Swine, Bolivia

**DOI:** 10.3201/eid1708.100769

**Published:** 2011-08

**Authors:** Maria Chiara Dell’Amico, Annalisa Cavallo, José Luis Gonzales, Sara Irene Bonelli, Ybar Valda, Angela Pieri, Higinio Segundo, Ramón Ibañez, Antonia Mantella, Filippo Bartalesi, Francesco Tolari, Alessandro Bartoloni

**Affiliations:** Author affiliations: Università degli Studi di Pisa, Pisa, Italy (M.C. Dell’Amico, F. Tolari);; Università degli Studi di Firenze, Firenze, Italy (A. Cavallo, S.I. Bonelli, A. Pieri, A. Mantella, F. Bartalesi, A. Bartoloni);; Laboratorio de Investigación y Diagnóstico Veterinario, Santa Cruz, Bolivia (J.L. Gonzales, Y. Valda, R. Ibañez);; Distrito de Salud Cordillera, Santa Cruz (H. Segundo)

**Keywords:** hepatitis E virus, viruses, humans, swine, ELISA, RT-PCR, phylogenetic analysis, genotype 3, Bolivia, dispatch

## Abstract

We determined the seroprevalence of hepatitis E virus (HEV) in persons in 2 rural communities in southeastern Bolivia and the presence of HEV in human and swine fecal samples. HEV seroprevalence was 6.3%, and HEV genotype 3 strains with high sequence homology were detected.

Hepatitis E virus (HEV) is the causative agent of epidemic and sporadic acute hepatitis in areas with poor sanitary conditions. The infection is endemic to southeast and central Asia, the Middle East, northern and western parts of Africa, and North America (*1*). Sporadic cases of HEV infection have also been reported in industrialized countries, usually associated with traveling in disease-endemic areas. Transmission of HEV occurs predominantly by the fecal–oral route, mostly through contaminated drinking water (*1*). Several animal sources of HEV have been identified (*2*). The first strain of animal origin was identified in 1997 in swine in the United States (*3*). Subsequently, several studies documented that swine are the largest reservoir of HEV (*4**–**7*).

Little data are available on HEV seroprevalence in countries in South America (*8*). The first study of HEV infection in Bolivia was a seroprevalence survey conducted in 1997 in rural southeastern Bolivia (*9*). The purposes of this study were to reassess HEV seroprevalence in humans in the same area and identify the virus in humans and swine.

## The Study

The survey was conducted in November–December 2006. We surveyed 2 rural communities in southeastern Bolivia: Bartolo in Hernando Siles Province, Department of Chuquisaca; and Casas Viejas in Vallegrande Province, Department of Santa Cruz.

The local economy is based on agriculture and animal farming. Persons live in close contact with animals, mainly swine, in the absence of adequate housing or fencing. Informed consent to obtain samples was obtained from all adults and from parents of minors participating in the survey. The study design, including its ethical aspects, was approved by the Bolivian Ministry of Health and the local health authorities.

Blood samples were taken from 236 persons (172 in Bartolo and 64 in Casas Viejas, age range 1–87 years). Individual fecal samples were obtained from 122 persons (90 in Bartolo and 32 in Casas Viejas, age range 3–62 years). Feces samples were combined into 22 pools (16 in Bartolo and 6 in Casas Viejas). Each pool consisted of 4–10 individual fecal samples from persons of the same age range. Individual fecal samples were taken also from 121 swine (67 in Bartolo and 54 in Casas Viejas, age range 2–12 months) and combined into 22 pools (13 in Bartolo and 9 in Casas Viejas).

Serum samples were tested for immunoglobulin (Ig) G against HEV. Samples from persons with fecal samples positive for HEV RNA were also tested for IgM against HEV by using commercial ELISA kits (HEV IgG/IgM; DIA.PRO Srl, Milan, Italy).

HEV RNA was detected by using reverse transcription PCR (RT-PCR) with 22 human and 22 swine fecal pools. Three grams of feces from each pool were homogenized in phosphate-buffered saline and centrifuged for 1 hour at 4°C. RNA was extracted by using the QIAamp Viral RNA Mini Kit (QIAGEN, Hilden, Germany). RT-PCR was performed in reduced reaction volumes of 25 µL instead of 50 µL (*10*). For human pools positive for HEV RNA, PCR was conducted with individual fecal samples that had been used in the pool.

For phylogenetic analysis, internal primer sequences were used to amplify isolates of human and swine HEV. The 348-nt sequence in open reading frame 2 of HEV isolates was analyzed and compared with corresponding regions of other known human and swine HEV strains available in GenBank. Sequences were aligned by using ClustalW2 (www.ebi.ac.uk/Tools/msa/clustalw2). Phylogenetic analysis was conducted by using MEGA5 (www.megasoftware.net). Geographic origin, identification code, and GenBank accession numbers of nucleotide sequences of the HEV strains used in the phylogenetic and sequence analyses are Japan, JJT-Kan (AB091394); Japan, JSN-Sap-FHo2C (AB200239); Japan, JSN-Sap (AB091395); Japan, JKK-Sap (AB074917); People’s Republic of China, swDQ (DQ279091); Japan, JRA1 (AP003430); Japan, HEJI4 (AB080575); United States, avian-HEV (EF206691); Japan, swJ8–5 (AB248521); People’s Republic of China, swCH31 (DQ450072); Japan, swJ12–4 (AB248522); India, Ind-sw01 (AY723745); Japan, HE-JA37 (AB220978); Japan, HE-JA04–1911 (AB248520); Japan, JMNG-Oki02C (AB236320); Japan, HE-JA41 (AB220979); Japan, swJ13–1 (AB097811); India, Yam67 (AF459438); India, Hyderabad (AF076239); and Mexico, Mexican strain (M74506).

A total of 15 (6.3%) of 236 serum samples were positive for HEV by IgG ELISA (Table). The prevalence of IgG against HEV (7%) was higher in persons in Bartolo than in persons in Casas Viejas (4.7%) (p = 0.5). Seroprevalence did not show a linear trend associated with age; the highest seroprevalence was in found for persons 41–50 years of age in both communities (median age of 15 HEV seropositive persons 45 years, range 2–87 years). None of the persons in these 2 groups had a history of jaundice. No sex-related differences in seroprevalence were observed in these communities (male participants 47%, female participants 53%).

**Table:** Prevalence of antibodies against hepatitis E virus in 236 human serum samples, by patient age, Bartolo and Casas Viejas, Bolivia

Patient age, y	No. positive/no. tested (% positive)			95% Confidence interval for total % positive
	Bartolo	Casas Viejas	Total	
1–5	2/18 (11)	0/5 (0)	2/23 (8.7)	1.1–28
6–10	0/26 (0)	0/12 (0)	0/38 (0)	0–9.2
11–20	1/35 (2.8)	0/7 (0)	1/42 (2.4)	0–12.6
21–30	1/25 (4)	1/6 (16.7)	2/31 (6.4)	0.8–21.4
31–40	1/19 (5.2)	0/6 (0)	1/25 (4.0)	0.1–20.3
41–50	5/18 (27.8)	0/3 (0)	5/21 (24.0)	8.2–47.1
51–60	1/19 (5.3)	0/10 (0)	1/29 (3.4)	0–17.8
>60	1/12 (8.3)	2/15 (13.3)	3/27 (11.0)	2.3–29.1
Total	12/172 (7)	3/64 (4.7)	15/236 (6.3)	3.6–10.3

Figure. Phylogenetic tree with alignments of 348-bp open reading frame 2 sequences from human and swine samples of hepatitis E virus (HEV), Bartolo and Casas Viejas, Bolivia, compared with sequences of various HEV isolates. The tree was constructed by using the neighbor-joining method and evaluated by using the interior branch test method with MEGA5 software (www.megasoftware.net). Percentage of bootstrap support is shown by values at the branch nodes of the tree. Only nodes with a bootstrap value >60% are labeled; these values are the result of resampling the data 1,000 times. Isolate names are followed by species of origin. Roman numerals indicate genotype. Avian HEV was used as the outgroup. Scale bar indicates nucleotide substitutions per site.

HEV-RNA was detected in 5 (22.7%) of 22 human fecal pools. All 31 fecal samples in the 5 HEV-positive pools were evaluated for HEV RNA. Four samples obtained from persons with an age range of 10–48 years were positive. All 4 amplification products were sequenced. HEV RNA was detected in 7 (31.8%) of 22 swine fecal pools (6 [46%] in Bartolo and 1 [11%] in Casas Viejas). Fecal samples from all 4 persons positive for HEV by PCR were negative for IgM and IgG against HEV by ELISA.

Phylogenetic analysis was performed on 11 amplification products obtained from 4 human and 7 swine fecal samples. Swine HEV sequences were closely related to human HEV sequences (76% nt and 92% aa homologies), and all belonged to HEV genotype 3. The amino acid homology among human HEV sequences was 96%. The phylogenetic tree produced from alignment of the 348-nt open reading frame 2 sequences is shown in the Figure.

## Conclusions

In a survey performed in 1997 in southeastern Bolivia, we found a seroprevalence of 7% for antibodies against HEV (*9*), a relatively high seroprevalence among young adults, and a low seroprevalence among children. This study, conducted 9 years later in the same area, showed a similar seroprevalence (6%) and age-dependent distribution of antibodies against HEV and identified HEV RNA in fecal samples from human and swine populations.

Sequence comparisons and phylogenetic analyses showed that swine HEV strains were closely related to human strains; all belonged to genotype 3. HEV was also detected on swine farms in South America (*11**,**12*). The high degree of nucleotide sequence homology observed suggests that swine could also be a major source of HEV in the area of our study, but we are uncertain whether these findings can be extrapolated to other areas of Bolivia. Absence of clinical signs in the swine studied is not an unexpected finding because swine naturally infected with HEV are usually asymptomatic (*13*).

With regard to humans, no history of jaundice in seropositive persons was reported. The 4 persons with positive HEV RNA results by RT-PCR but negative IgM and IgG results by ELISAs were asymptomatic at sample collection and had no signs or symptoms of hepatitis 3 months after testing. We cannot explain the negative HEV serologic results for these persons. This finding could be attributed to early infection, transient intestinal virus passage, or low accuracy of assays used (*14*). Apparent limited illness associated with HEV infection in humans may be caused by attenuated virulence of genotype 3 strains (*15*). Additional studies are needed to define illness associated with HEV infection in humans and determine whether HEV infection is present in other animal species, particularly rodents, and their role in transmitting this virus (*13*).

## References

[1] Mushahwar IK. Hepatitis E virus: molecular virology, clinical features, diagnosis, transmission, epidemiology, and prevention. J Med Virol. 2008;80:646–58. 10.1002/jmv.21116 18297720

[2] Meng XJ. Hepatitis E virus: animal reservoirs and zoonotic risk. Vet Microbiol. 2010;140:256–65. 10.1016/j.vetmic.2009.03.017 19361937 PMC2814965

[3] Meng XJ, Purcell RH, Halburb PG, Lehman JR, Webb DM, Tsareva TS, A novel virus in swine is closely related to the human hepatitis E virus. Proc Natl Acad Sci U S A. 1997;94:9860–5. 10.1073/pnas.94.18.9860 9275216 PMC23282

[4] Lu L, Li C, Hagedorn CH. Phylogenetic analysis of global hepatitis E virus sequences: genetic diversity, subtypes and zoonosis. Rev Med Virol. 2006;16:5–36. 10.1002/rmv.482 16175650

[5] Kabrane-Lazizi Y, Fine JB, Elm J, Glass GE, Higa H, Diwan A, Evidence for widespread infection of wild rats with hepatitis E virus in the United States. Am J Trop Med Hyg. 1999;61:331–5. 10463689 10.4269/ajtmh.1999.61.331

[6] Favorov MO, Kosoy MY, Tsarev SA, Childs JE, Margolis HS. Prevalence of antibody to hepatitis E virus among rodents in the United States. J Infect Dis. 2000;181:449–55. 10.1086/315273 10669325

[7] Arankalle VA, Goverdhan MK, Banerjee K. Antibodies against hepatitis E virus in Old World monkeys. J Viral Hepat. 1994;1:125–9. 10.1111/j.1365-2893.1994.tb00111.x 8790567

[8] Bortoliero AL, Bonametti AM, Morimoto HK, Matsuo T, Reiche EM. Seroprevalence for hepatitis E virus (HEV) infection among volunteer blood donors of the regional blood bank of Londrina, state of Paraná, Brazil. Rev Inst Med Trop Sao Paulo. 2006;48:87–92. 10.1590/S0036-46652006000200006 16699630

[9] Bartoloni A, Bartalesi F, Roselli R, Mantella A, Caceres Arce C, Paradisi P, Prevalence of antibodies against hepatitis A and E viruses among rural populations of the Chaco region, south-eastern Bolivia. Trop Med Int Health. 1999;4:596–601. 10.1046/j.1365-3156.1999.00457.x 10540299

[10] Meng XJ, Purcell RH, Halbur PG, Lehman JR, Webb DM, Tsareva TS, A novel virus in swine is closely related to the human hepatitis E virus. Proc Natl Acad Sci U S A. 1997;94:9860–5. 10.1073/pnas.94.18.9860 9275216 PMC23282

[11] Munné MS, Vladimirsky S, Otegui L, Castro R, Brajterman L, Soto S, Identification of the first strain of swine hepatitis E virus in South America and prevalence of anti-HEV antibodies in swine in Argentina. J Med Virol. 2006;78:1579–83. 10.1002/jmv.20741 17063523

[12] dos Santos DR, Vitral CL, de Paula VS, Marchevsky RS, Lopes JF, Gaspar AM, Serological and molecular evidence of hepatitis E virus in swine in Brazil. Vet J. 2009;182:474–80. 10.1016/j.tvjl.2008.08.001 18805029

[13] Meng XJ. Hepatitis E virus: animal reservoirs and zoonotic risk. Vet Microbiol. 2010;140:256–65. 10.1016/j.vetmic.2009.03.017 19361937 PMC2814965

[14] Yamamoto H, Li TC, Koshimoto C, Ito K, Kita M, Miyashita N, Serological evidence for hepatitis E virus infection in laboratory monkeys and pigs in animal facilities in Japan. Exp Anim. 2008;57:367–76. 10.1538/expanim.57.367 18633159

[15] Halbur PG, Kasorndorkbua C, Gilbert C, Guenette D, Potters MB, Purcell RH, Comparative pathogenesis of infection of pigs with hepatitis E viruses recovered from a pig and a human. J Clin Microbiol. 2001;39:918–23. 10.1128/JCM.39.3.918-923.2001 11230404 PMC87850

